# SWI/SNF-deficient undifferentiated/rhabdoid carcinoma of the gallbladder carrying a POLE mutation in a 30-year-old woman: a case report

**DOI:** 10.1186/s13000-021-01112-4

**Published:** 2021-06-12

**Authors:** Tiemo S. Gerber, Abbas Agaimy, Arndt Hartmann, Michael Habekost, Wilfried Roth, Albrecht Stenzinger, Peter Schirmacher, Beate K. Straub

**Affiliations:** 1 Institute of Pathology, University Medical Center Mainz, Langenbeckstraße 1, 55131 Mainz, Germany; 2 Institute of Pathology, Erlangen University Hospital, Erlangen, Germany; 3 Department of General- and Visceral Surgery, Agaplesion Markus Krankenhaus, Frankfurt am Main, Germany; 4 Institute of Pathology, University Hospital Heidelberg, Heidelberg, Germany

**Keywords:** Case report, SWI/SNF-deficiency, Gallbladder carcinoma, SMARCA4, Rhabdoid

## Abstract

**Background:**

Undifferentiated carcinoma of the biliary tract are highly aggressive malignancies. In other organs, a subgroup of undifferentiated carcinoma related to SWI/SNF complex–deficiency have been described.

**Case presentation:**

A 30-year-old woman presented with rising inflammatory markers (C-reactive protein (CRP)). Ultrasound examination revealed a large tumor of the liver. A computed tomography scan was performed and was primarily interpreted as a tumor-forming liver abscess, possibly caused by gallbladder perforation. Subsequent liver segment resection was performed. Microscopic examination showed an undifferentiated carcinoma with rhabdoid morphology and prominent inflammatory infiltrate in the gallbladder base. With SWI/SNF immunohistochemistry, intact expression of SMARCB1, SMARCA4, ARID1A, but loss of SMARCA2 and PBRM1 was detected. Next-generation-sequencing detected *KRAS*, *PBRM1* and *ARID1B* mutations, a deleterious splice-site mutation in the *POLE*-gene and a mutation in the *TP53*-gene.

**Conclusions:**

We were able to demonstrate loss of SMARCA2 expression and mutations characteristic of an SWI/SNF-deficient carcinoma in a tumor derived from the gallbladder. This is the first reported case of an undifferentiated carcinoma with rhabdoid features in the gallbladder carrying a *POLE* mutation and SWI/SNF-deficiency of *PBRM1* and *SMARCA2*.

**Supplementary Information:**

The online version contains supplementary material available at 10.1186/s13000-021-01112-4.

## Background

Undifferentiated carcinomas (UC) of the gallbladder are malignant neoplasms with poor prognosis, accounting for 3.4 % of all gallbladder carcinomas (GBC) with a median survival of 7.3 months and a mean age of the patients of 67 years. UC most often presents as a polypoid mass of a median size of 5 cm in radiologic and pathologic gross examination. The morphology of UC may be variable, varying from small cell type, pleomorphic cell type, to pseudosarcomatous type. [1]

Neoplasms of the gastrointestinal tract with rhabdoid features are distinctive, highly aggressive tumors defined by rhabdomyoblast-like morphology but without evidence for rhabdomyosarcomatous differentiation. Most patients diagnosed with such tumors die within one year, with a mean survival of 4 months. [2, 3]

SNF (sucrose nonfermenting) and SWI (switch) genes encode subunits of large protein complexes that exert a crucial role as global regulators of transcription through ATP-dependent chromatin remodeling. SWI/SNF complexes contain > 20 genes including *SMARCA2*, *SMARCA4*, *SMARCC1*, *SMARCC2*, *SMARCB1*, *ARID1A*, *PBRM1* among others. SWI/SNF complexes play critical roles in cancer cell differentiation and induce aberrant activation of Hedgehog signaling as well as alterations in cellular adhesion and motility. [4] *SMARCB1* (*INI1*, *SNF5/BAF47*) was the first member of the SWI/SNF-complex found to be mutated in cancer: families with heterozygous germline *SMARCB1* mutations were described with extrarenal rhabdoid tumors with loss of expression of SMARCB1 presenting already in early childhood. [5] Apart from rhabdoid tumors, meanwhile, also in carcinomas, aberrations of SWI/SNF complex proteins have been frequently observed. Commonly mutated SWI/SNF subunits include SMARCA4 inactivation in undifferentiated thoracic malignancies and small cell carcinoma of the ovary, hypercalcemic type [6, 7] as well as poorly differentiated sinonasal carcinoma [8], *SMARCB1* inactivation in epithelioid sarcoma [9, 10], *PBRM1* truncating mutations in clear cell renal cell carcinoma [11], and inactivating mutations of members of the *ARID* family in many different cancer types, including cholangiocarcinoma [12]. In dedifferentiated and undifferentiated endometrial carcinoma, 18–66 % of tumors show defective alterations of SWI/SNF complex proteins, of which patients with *POLE*-mutated cancers demonstrate better prognosis [13–15]. *ARID1A* and *ARID1B*, member of the SWI/SNF complexes, are involved in the regulation of transcription, DNA repair, and the epigenetic landscape via the DNA polymerase POLE [16, 17]. The genetic alterations may be somatic or be linked with germline mutations, resulting in the familial clustering of these tumors. [18] Concerning gastrointestinal undifferentiated carcinoma, loss of SWI/SNF complex proteins has already been described in UC of the stomach, colon, vermiform appendix, duodenum, and the pancreas, often leading to rhabdoid features [19].

We here report a rare case of an undifferentiated carcinoma of the gallbladder in a young woman with unique molecular changes.

## Case presentation

We present the case of a 30-year-old woman with diarrhea and acute onset of upper abdominal pain. She had no personal or family history of cancer. There weren`t any previous interventions known. Subsequent ultrasound examination revealed a strongly echogenic lesion in liver segment VI. A computed tomographic scan of the abdomen performed with the intravenous administration of contrast medium showed a pericholecystic fluid collection measuring 12 cm x 9 cm and an irregular thickening of the gallbladder wall, so the presumptive radiological diagnosis of acute cholecystitis complicated by gallbladder perforation and abscess formation was made. Clinical differential diagnoses included liver abscess, cystic echinococcosis, and neoplastic disease, so following tumor board decision, the patient was admitted to open procedure resection with cholecystectomy and liver segment V / VI resection of the lesion.

**Fig. 1:**
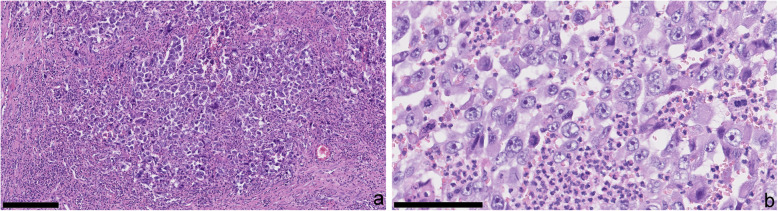
Histopathology of the undifferentiated carcinoma with rhabdoid features.(**a**) Morphology shows a highly pleomorphic, highly proliferative carcinoma with, eosinophilic cytoplasm, hyperchromatic nuclei and prominent nucleoli as well as admixed abundant neutrophilic granulocytes. H&E staining; bar: 250 μm. (**b**) high power aspect of the carcinoma (H&E staining; bar: 100 μm)

**Fig. 2:**
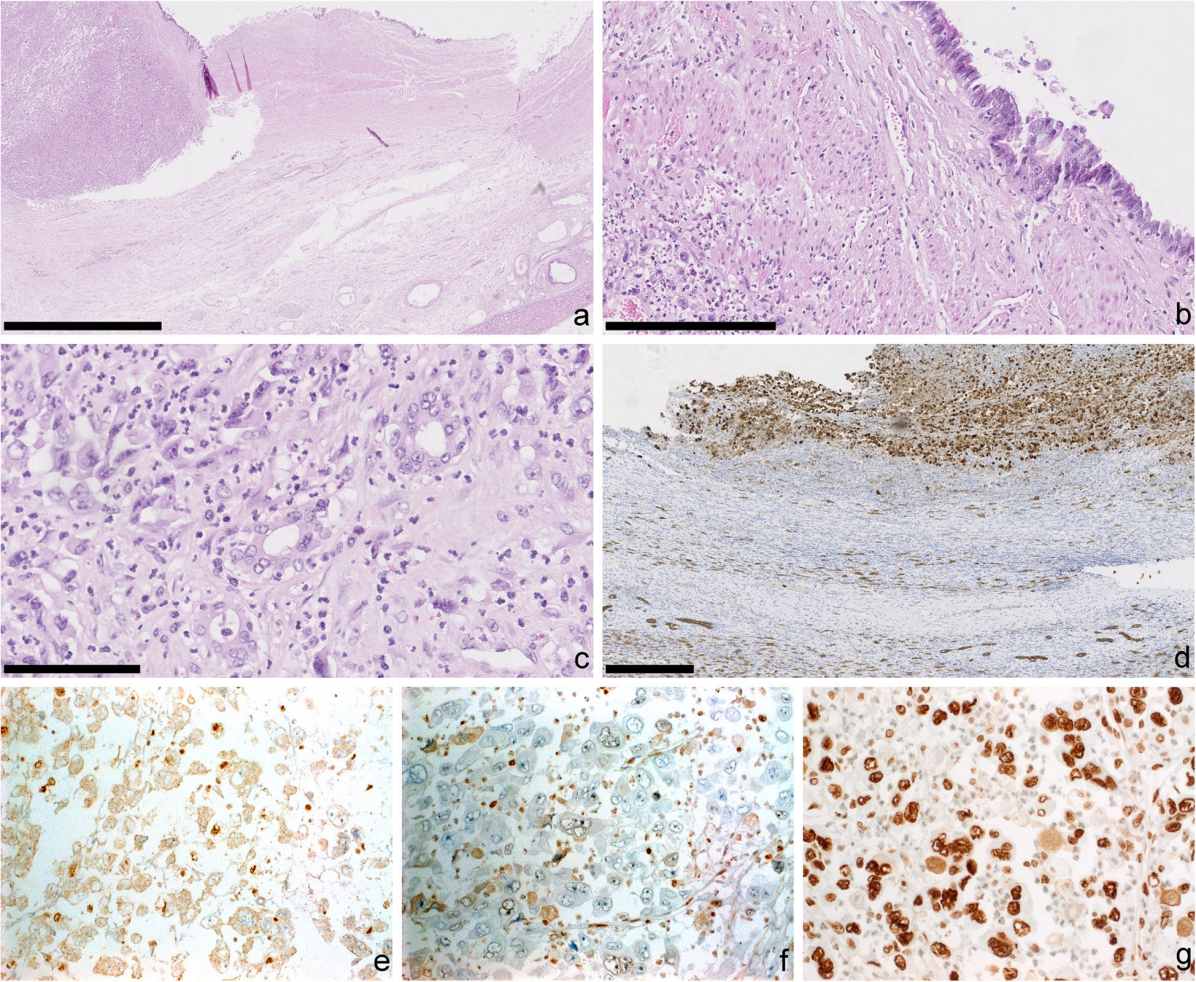
Glandular differentiation of the undifferentiated carcinoma with rhabdoid features. (**a**) Tumor with neighbored biliary intraepithelial neoplasia of the gallbladder and liver. (H&E staining; bar: 2,5 mm). (**b**) High power aspect of the biliary intraepithelial neoplasia (H&E staining; bar: 250 μm). (**c**) Gland formation with atypical nuclei (H&E staining; black bar: 100 μm). (**d**) Immunohistochemistry for cytokeratin 8 with positive cytoplasmic staining in the tumor cells (top). For internal positive control see bile ducts (bottom) (bar: 500 μm). (**e**) Immunohistochemistry for PBRM1 without nuclear staining in the tumor cells (negative). Note nuclear staining in inflammatory cells. (**f**) Loss of nuclear SMARCA2 expression by immunohistochemistry in tumor cells. (**g**) Immunohistochemistry for SMARCA4 with retained expression. (e-g: 400 x magnification)

At gross examination, liver resection showed a tumor-forming lesion of 13.5 cm in size with an irregular, grey surface, with partly crumbling and suppurating, partly indurated texture in close proximity to the gallbladder. The gallbladder was of 6.7 cm length with a thickened wall of up to 3 cm and contained yellow concretions. Histological examination revealed a highly pleomorphic, highly proliferative, overtly malignant tumor with hyperchromasia, atypical mitotic figures, eosinophilic cytoplasm, and prominent nucleoli (Fig. 1). Besides, geographic necrosis, multinucleated tumor giant cells and abundant neutrophilic infiltrate were present. The tumor infiltrated the gallbladder wall and adjacent adipose tissue, as well as the liver. In the proximity of the tumor, the gallbladder epithelium displayed atypia with an increased nuclear/cytoplasmic ratio, hyperchromasia, and loss of nuclear polarity, characteristic of high-grade biliary intraepithelial neoplasia. Focal gland formation was observed, reminiscent of an adenocarcinoma (Fig. 2). Immunohistochemistry of the carcinoma revealed positivity solely for cytokeratin 8 and vimentin. Immunohistochemical stains against TTF-1, HepPar1, PAX8, OCT3/4, CEA, BerEp4, estrogen receptor, cytokeratins 7 and 20, as well as CDX2 and ErbB2 were negative. Ki-67-proliferation rate (MIB-1 labeling index) was over 95 %. PBRM1- and SMARCA2-expression were lost in the carcinoma but retained in the associated high-grade biliary intraepithelial neoplasia. SMARCA4, SMARCB1 (INI-1), and ARID1A were retained. PDL-1 was strongly expressed in 80 % of tumor cells as well as in some inflammatory cells. Next-generation-sequencing with the Oncomine-Comprehensive Assay v3 detected a *KRAS* mutation (p.Gly12Asp; allele frequency 29,5 %), a deleterious splice-site mutation in the *POLE*-gene (NM_006231:c.4729-1G > A; allele frequency 7,3 %) and a *TP53* splice site mutation (allele frequency 17,9 %). Besides, a variant of unknown significance in the *SMARCA4*-gene (NM_003072:p.Leu1063Val; allele frequency 14,5 %) was discovered. In addition, *PBRM1* and *ARID1B* mutations were detected, among others (see Table 1 and Supplemental 1). The mutations were also verified using another NGS panel (Illumina TruSight Tumor 500-Panel) using two different tumor areas. A comparative analysis of biliary intraepithelial neoplasia and invasive tumor was not possible due to the spatial proximity and tumor spill-over. The mutational burden was increased (12.53 and 11.01 mutations per megabase) [20]. The tumor was microsatellite stable and showed tumor heterogeneity.

**Table 1:** Mutations detected with Illumina TruSight Tumor 500 panel. Concerning all mutations as well as copy number alterations see Supplemental Table 1

*Gene*	*Mutation*	*Location 1*	*Location 2*	*Variant cDNA*
***KRAS***	p.Gly12Asp	**24.8 %**	**61.6 %**	c.35G > A
***TP53***	p.spl?	**13.7 %**	**32.6 %**	c.993 + 1G > T
***POLE***	p.spl?	**4.8 %**	**16.5 %**	c.4729-1G > A
***PBRM1***	p.Leu526Val	**23.9**	**49.1**	c.1576T > G
***ARID1B***	p.Ser1869Cys	/	**67.4**	c.5606 C > G

In the postoperative course, elevated serum CRP levels declined under systemic intravenous antibiotics. The patient died within ten months of the diagnosis.

## Discussion

Although two cases of carcinomas of the gallbladder with rhabdoid features have already been described [21, 22], this case report describes the first report of undifferentiated carcinoma of the gallbladder with rhabdoid features and SWI/SNF-deficiency. Pathological examination revealed complete dedifferentiation of the tumor, although focal gland formation, retained epithelial marker expression, and high-grade biliary intraepithelial neoplasia helped us to conclude an origin from the gallbladder epithelium. From morphology alone, initial differential diagnostic considerations included e.g. extrarenal rhabdoid tumor, yet loss of expression of SMARCB1 (INI1) was not observed. Another important differential diagnosis was sarcoma not otherwise specified. Biliary intraepithelial neoplasia and focal glandular formation favored the diagnosis of an epithelial origin. Rhabdoid carcinomas may occur in different anatomical locations including the gastrointestinal tract, but they share a similar molecular origin, which results in SWI/SNF-deficiency [3, 23]. Typically, SWI/SNF-deficiency may be restricted to the undifferentiated part of the carcinoma. In a thorough review of the literature, Agaimy and coauthors [2] revealed a total of 39 carcinomas of the digestive tract with similar rhabdoid features in the stomach, colon, small bowel, and distal esophagus. 64 % of patients were 60 years of age or older, which is in contrast to our case of a young, 30-year-old women.

Carcinoma of the gallbladder is a rare biliary tract malignancy in western countries with substantial geographic distribution variation. The development of carcinoma of the gallbladder is associated with chronic inflammation of the gallbladder, dietary factors, and female gender. This malignancy is a multifactorial disorder involving multiple genetic alterations, most commonly *KRAS*-, *TP53*- and *c-erb-B2*-genes. [24] Within our case, besides SWI/SNF-deficiency, mutations in the *KRAS*-, the *POLE*-gene, and the *TP53*-gene were detected, but no aberration in *c-erb-B2*. Mutations in *POLE*, which encodes proofreading DNA Polymerase, are often associated with high mutational burden, thus leading to an ultramutated phenotype [25]. However, causality for tumor ultramutation has only been described for exonuclease domain mutations (EDM). Pathogenic *POLE* EDM typically display characteristic genomic alterations and are therefore proposed as a distinct clinical entity. [26]. In our case, the role of the *POLE* mutation and its pathogenicity remains unclear. The young age of the patient may point to a germline predisposition mutation, but such analyses have not been performed. The mutation in *TP53* we detected in the tumor has been described to be often present as germline mutation. Normal tissue has not been analysed for ethical reasons. The polymerase proofreading-associated polyposis syndrome (PPAP) with missense mutations in the *POLE*- or *POLD1*-gene was recently described as cause of a new polyposis syndrome with development of colorectal cancers [27]. However, other tumor types like endometrial carcinoma, gastric cancer, breast cancer, melanomas and glioblastomas were also described to occur in this syndrome [28]. In our case, the prominent inflammatory infiltrates together with strong PDL1-expression may open the possibility for therapy with checkpoint inhibitors.

## Conclusions

We here present the unique case of an undifferentiated rhabdoid carcinoma of the gallbladder with *POLE* gene mutation and SWI/SNF-deficiency. The diagnosis was made based on a precursor lesion of the gallbladder epithelium and the presence of residual glandular tumor structures as well as a loss of SMARCA2 and mutations in *PBRM1* and *ARID1B*. We hope that the case demonstrated here will be useful to deepen the understanding of SWI/SNF-deficient carcinomas of the gastrointestinal tract.

## Supplementary Information

**Additional file 1.**

## Data Availability

Data sharing does not apply to this article as no datasets were generated or analyzed during the current study.

## Abbreviations

EDM: Exonuclease domain mutations
GBC: Gallbladder carcinoma
UC: Undifferentiated carcinoma
SWI/SNF: SWItch Sucrose Non-Fermentable
PPAP: Polymerase proofreading-associated polyposis syndrome
