# Supplementary material for: SWI/SNF-deficient undifferentiated/rhabdoid carcinoma of the gallbladder carrying a POLE mutation in a 30-year-old woman: a case report
Source: Diagn Pathol. 2021 Jun 12;16:52. doi: 10.1186/s13000-021-01112-4 (PMC8196506; doi:10.1186/s13000-021-01112-4)
Supplement: Supplementary file 1 — Additional file 1. [file 13000_2021_1112_MOESM1_ESM.docx]

| ***Gene*** | ***Mutation*** | ***cDNA*** | ***NM-Number*** | ***Location 1*** | ***Location 2*** |
| --- | --- | --- | --- | --- | --- |
| MYC | p.Met125Leu | c.373A>C | NM_002467 | 30.5 | 15.3 |
| FANCA | p.Asp902Glu | c.2706C>G | NM_000135 | 42.8 | 54.9 |
| SMARCA4 | p.Leu1063Val | c.3187T>G | NM_003072 | 12.9 | 37.7 |
| ZFHX3 | p.Ala514Thr | c.1540G>A | NM_006885 | 48.8 | 46.3 |
| CARD11 | p.Ala1047Thr | c.3139G>A | NM_032415 | 50.8 | 49.5 |
| TOP2A | p.Lys1289Glu | c.3865A>G | NM_001067 | 46.4 | 49.7 |
| RANBP2 | p.Asn834Lys | c.2502C>G | NM_006267 | 7.7 | 15.7 |
| KAT6A | p.Arg1086Cys | c.3256C>T | NM_006766 | 56 | 72.2 |
| RET | p.Ile578Val | c.1732A>G | NM_020630 | / | 58.8 |
| RARA | p.Pro30Ala | c.88C>G | NM_000964 | / | 50.4 |
| ICOSLG | p.Ile273Val | c.817A>G | NM_015259 | / | 33 |
| MED12 | p.Met406Val | c.1216A>G | NM_005120 | / | 68.6 |
| MCL1 | p.Ile237Val | c.709A>G | NM_021960 | 57.7 | 67.9 |
| SPTA1 | p.Met2209Leu | c.6625A>T | NM_003126 | 46.5 | 41.9 |
| PIK3C2B | p.Ser553Gly | c.1657A>G | NM_002646 | 43.2 | 38.8 |
| LRP1B | p.Gly1691Ala | c.5072G>C | NM_018557 | 53.5 | 68.6 |
| INHA | p.Pro253Leu | c.758C>T | NM_002191 | 52.2 | 45.8 |
| EPHA5 | p.Gly30Cys | c.88G>T | NM_004439 | / | 71 |
| CSF1R | p.Ser930Cys | c.2788A>T | NM_005211 | / | 47 |
| GRM3 | p.Leu140Phe | c.418C>T | NM_000840 | / | 14 |
| BRAF | p.Gly15Ser | c.43G>A | NM_004333 | / | 16.7 |
| ZNF703 | p.Ser20_Gly21dup | c.43_44insGCAGCG | NM_025069 | / | 34.8 |
| AXIN2 | p.Arg357His | c.1070G>A | NM_004655 | / | 40 |

**Supplemental Table 1: Mutations (continued) detected with Illumina TruSight Tumor 500 panel**

| ***Gene*** | ***RefSeq-ID*** | ***Location 1*** | ***Location 2*** | ***Cytoband*** |
| --- | --- | --- | --- | --- |
| ***MYC*** | NM_002467 | **/** | **Low-Level Amplification** | 8q24.21 |
| ***ZNF703*** | NM_025069 | **/** | **Whole-Gene Deletion** | 8p11.23 |
| ***MET*** | NM_001127500 | **/** | **Low-Level Amplification** | 7q31.1 |
| ***LAMP1*** | NM_005561 | **/** | **Low-Level Amplification** | 13q34 |
| ***CDK4*** | NM_000075 | **/** | **Low-Level Amplification** | 12q14.1 |
| ***ERBB3*** | NM_001982 | **/** | **Low-Level Amplification** | 12q13.2 |
| PAK1,C11orf30 | | **/** | **Region Gain** | 11q13.5-11q13.5 |
| CCND1,FGF3,FGF19,FGF4 | | **/** | **Region Gain** | 11q13.3-11q13.3 |
| CDKN2A,CDKN2B,CDKN2A,MLLT3 | | **/** | **Region Loss** | 9p21.3-9p21.3 |

**Supplemental Table 2: Genes with copy number alterations.**
